# Dermoglandular Rotation Flaps for Breast-Conserving Therapy: Aesthetic Results, Patient Satisfaction, and Morbidity in Comparison to Standard Segmentectomy

**DOI:** 10.1155/2014/152451

**Published:** 2014-11-04

**Authors:** Ursula Hille-Betz, Bernhard Vaske, Helga Henseler, Philipp Soergel, Sudip Kundu, Lars Makowski, Sophia Schelcher, Sebastian Wojcinski, Peter Hillemanns

**Affiliations:** ^1^Department of Obstetrics and Gynecology, Hannover Medical School, 30625 Hannover, Germany; ^2^Institute of Medical Biometry and Informatics, Hannover Medical School, 30625 Hannover, Germany; ^3^Department of Plastic, Hand and Reconstructive Surgery, Hannover Medical School, 30625 Hannover, Germany

## Abstract

We compared a dermoglandular rotation flap (DGR) in the upper inner, lower inner, and upper outer quadrant regarding similar aesthetic results, patient satisfaction, and comfort after breast-conserving therapy with standard segmentectomy (SE). Between 2003 and 2011, 69 patients were treated with breast-conserving surgery using DGR for cancers with high tumor-to-breast volume ratios or skin resection in the three above mentioned quadrants; 161 patients with tumors in the same quadrants were treated with SE. The outcome of the procedures was assessed at least 7 months after completed radiation therapy using a patient and breast surgeon questionnaire and the BCCT.core software. Symmetry, visibility of the scars, the position of the nipple-areola complex, and the appearance of the treated breast were each assessed on a scale from 1 to 4 by an expert panel and by the patients. Univariate and multivariate analysis were used to evaluate the relationship between patient-, tumor-, and treatment-dependent factors and patient satisfaction. 94.2% of the patients with rotation flaps and 83.5% of the patients with lumpectomy were very satisfied with the cosmetic appearance of their breast. Younger patient age was significantly associated with a lower degree of satisfaction. DGR provides good cosmetic results compared with SE and shows high patient satisfaction despite longer scarring and higher median resection volume.

## 1. Introduction

Breast-conserving surgery combined with postoperative radiotherapy is currently the standard treatment for the majority of women with breast cancer, and the value of this “conservative treatment” for small tumors is beyond question [1, 2]. Many patients treated with conservative surgery present with a good aesthetic result, especially patients undergoing standard segmentectomy (SE), even if the breast volume is small, due to the minimal amount of tissue excised [3].

However, the popularity of breast-conserving surgery even in larger tumors over the last 4 decades has increased the prevalence of adverse aesthetic results. Some authors presented classifications of these deformities and suggested reconstructive techniques to improve the aesthetic outcome [4–7]. Independently of the suggested classification, concerns remain after breast-conserving therapy: the distortion and dislocation of the nipple-areola complex and localized tissue insufficiency (skin deficiency, subcutaneous deficiency, or both) [8]. Cosmetic outcome is influenced by several factors such as resection volume, skin resection, radiation therapy, breast size, and tumor location. Volume displacement with recruiting and transposing local glandular or dermoglandular flaps into the resection site is one method for preventing visible deformity leading to an unacceptable cosmetic result [9–11].

The dermoglandular rotation technique can be used to fill a partial mastectomy defect in the upper or lower inner quadrant [9, 10] and for tumors located in the upper outer quadrant [12]. However, since the dermoglandular flaps technique, especially in the upper outer quadrant, has been well described [13], a case series focusing on postoperative aesthetic evaluation, patient satisfaction, and morbidity compared to standard segmentectomy has not been published. This study aims at comparing a dermoglandular rotation flap (DGR) in the upper inner, lower inner, and upper outer quadrant with SE.

## 2. Patients and Methods

Before any study activity, the ethical committee of our institution approved the study design. Written informed consent was also obtained from all patients who participated in the study.

### 2.1. Patients

We searched our database of patients with breast cancer and identified a series of 230 women with tumors in the upper inner, upper outer, and lower inner quadrants between 2003 and 2011 who had been treated with dermoglandular rotation flaps (69) or standard lumpectomy (161). The patients were invited for an examination of the breast and agreed to participate in this study, which was carried out at the Breast Care Unit at Hannover Medical School.

### 2.2. Oncoplastic Techniques

All dermoglandular rotation flaps were performed in a standard position with 45-degree elevation of the upper part of the body. All patients had preoperative markings in a standing position before the operation. A segmentectomy up to quadrantectomy through a radial skin incision that approached the nipple-areolar complex (NAC) tangentially was performed. The overlying skin was removed with full-thickness fibroglandular resection when oncologically necessary or to avoid excessive redundant skin.

**Figure 1:**
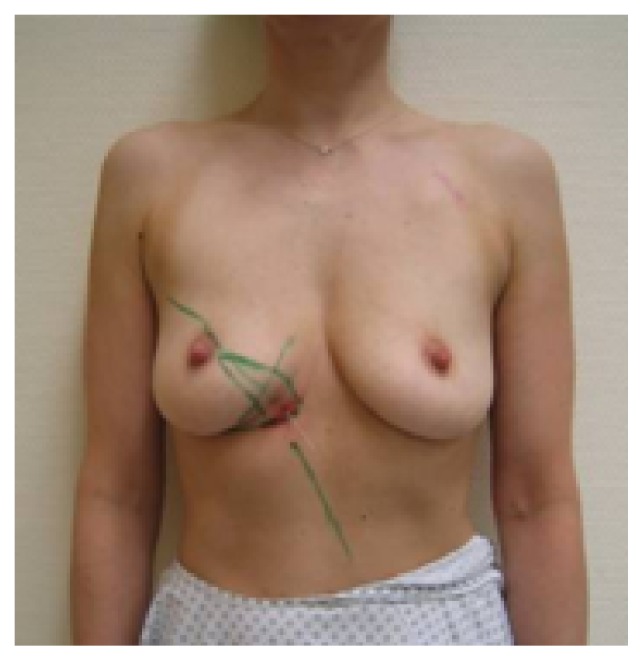
Patient of 43 years, ypT0pN0 resection volume 30 g, cup 75C.

**Figure 2:**
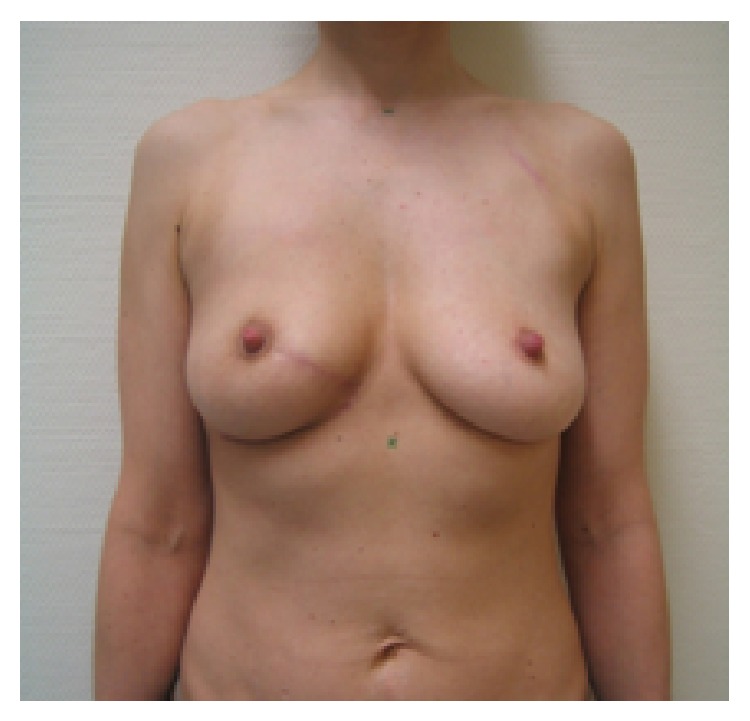
19-month follow-up; scar length 25 cm; patient was very satisfied with cosmetic result; BCCT.core: good; the scar visibility was rated as* slightly visible* by the patient and the experts, and the volume discrepancy was rated* slight* by the patient and* not at all* by the experts.

**Figure 3:**
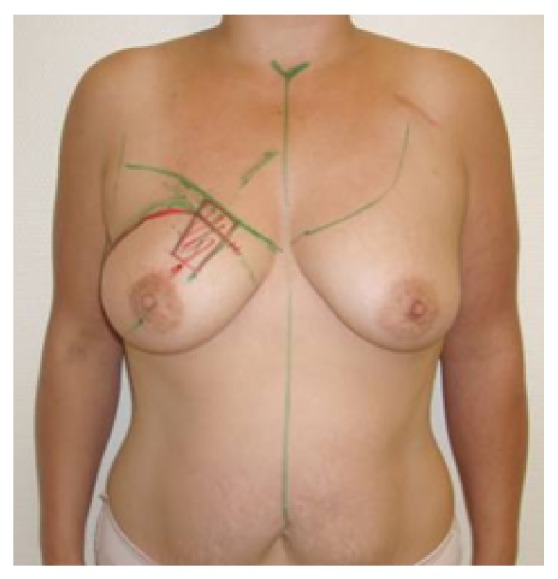
Patient of 46 years, pT2pN2, resection volume 55 g, cup 80C, frontal view.

**Figure 4:**
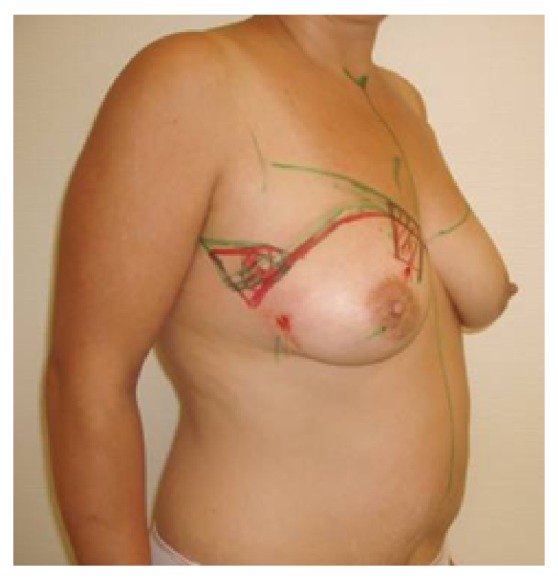
Same patient, oblique view.

**Figure 5:**
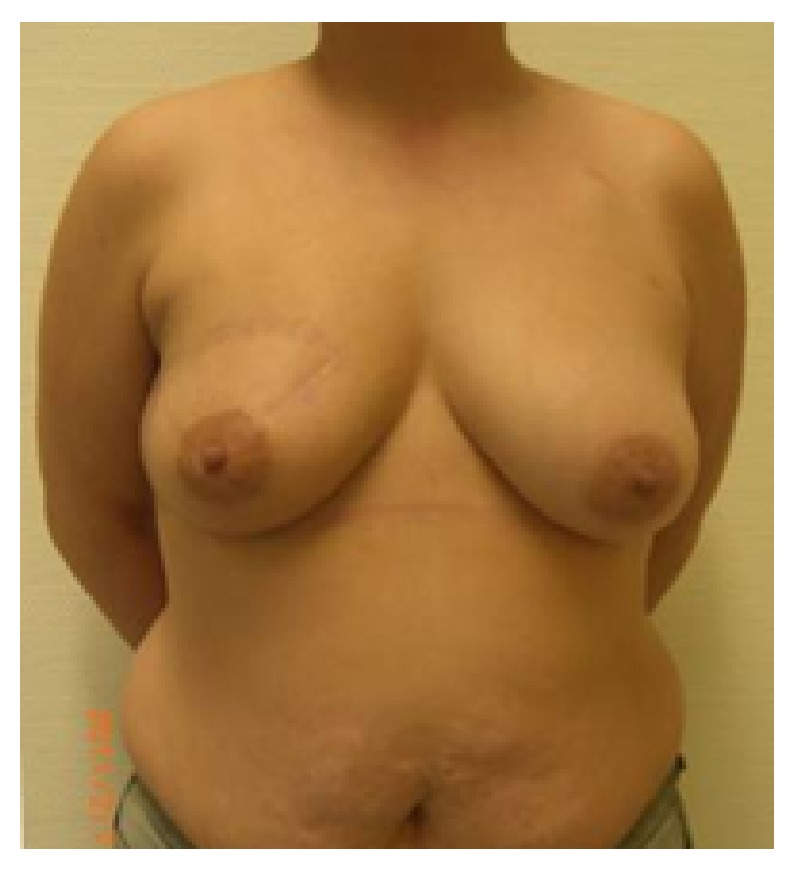
Same patient, 19-month follow-up; scar length 25 cm; BCCT.core: excellent. The patient was very satisfied with the cosmetic result. The scar visibility was rated as* very visible* by the patient and* moderately visible* by the experts. The volume discrepancy was rated as* slight* by the patient and* not at all* by the experts; frontal view.

**Figure 6:**
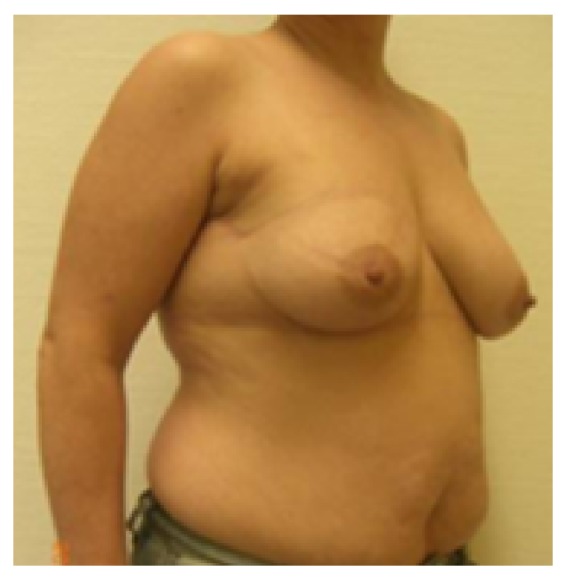
Same patient at 19-month follow-up, oblique view.

**Figure 7:**
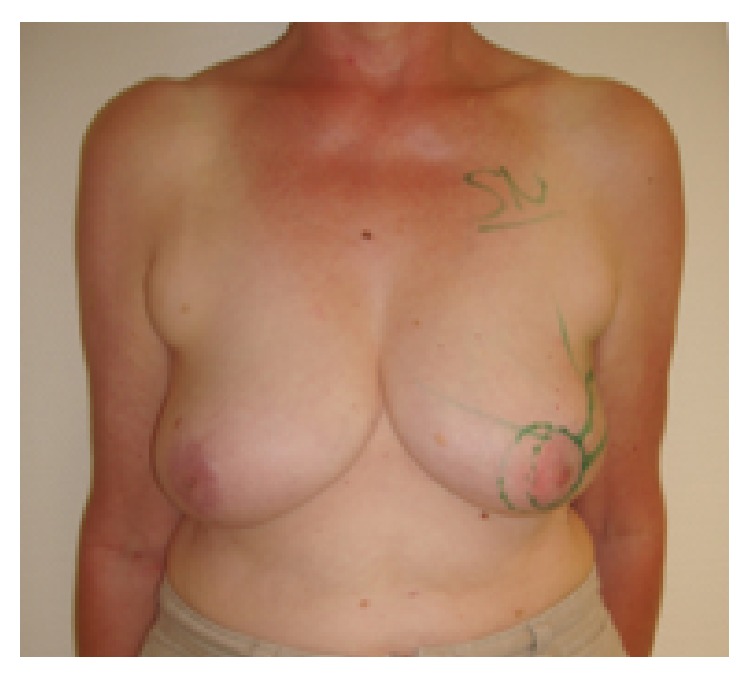
Patient of 69 years, pT2, pN0, resection volume 61 g, cup 80B, frontal view.

**Figure 8:**
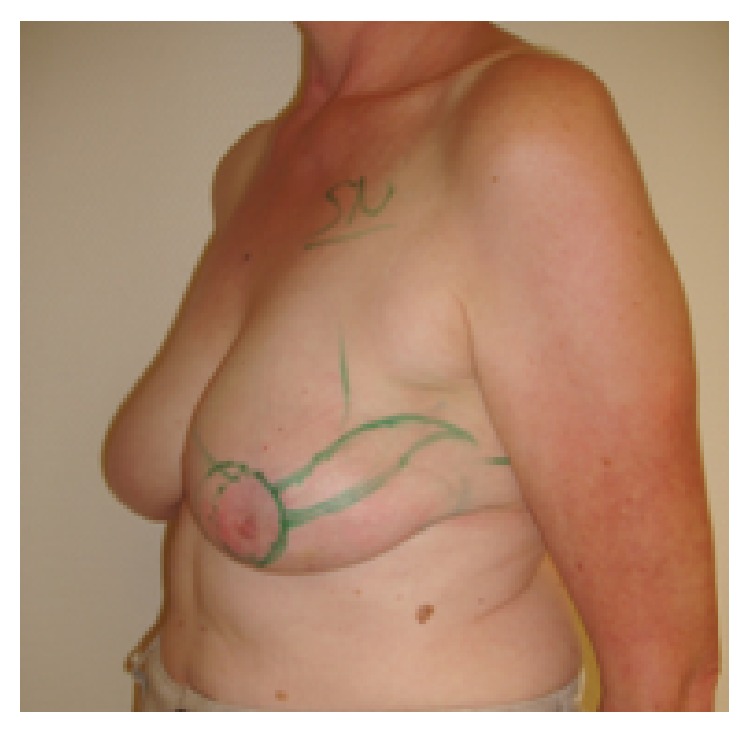
Same patient, oblique view.

**Figure 9:**
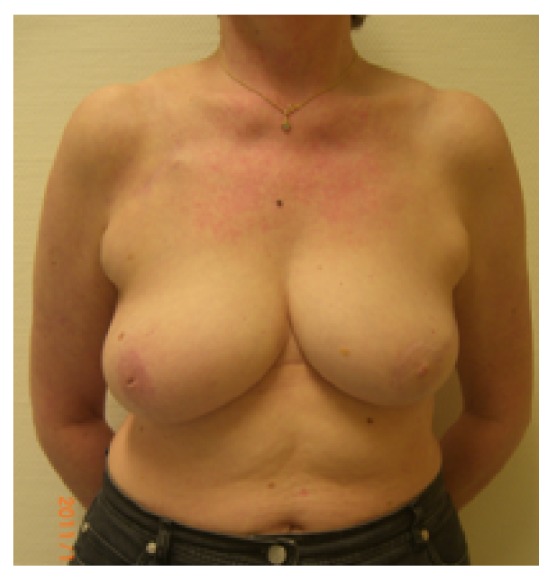
Same patient, 18-month follow-up, frontal view; scar length 27 cm; BCCT.core: fair. The patient was very satisfied with the cosmetic result. The scar visibility was rated as* slightly visible* by the patient and* moderately visible* by the experts. The volume discrepancy was rated as* slight* by the patient and experts.

**Figure 10:**
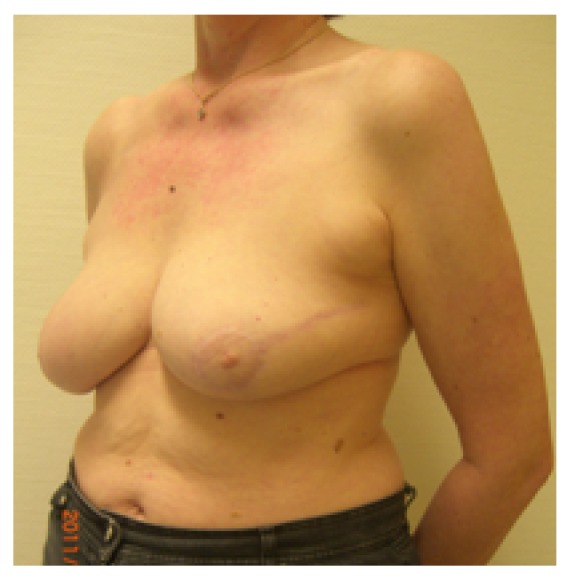
Same patient at 18-month follow-up, oblique view.

In tumors in the lower inner quadrant, the skin incision was extended along the submammary fold, and the mammary gland was mobilized on the pectoralis muscle (Figures 1 and 2) [10, 12]. In tumors in the upper inner quadrant, the incision led toward the lateral border of the breast where a mirror inverted triangle of skin was removed (Figures 3, 4, 5, and 6). The mobilization of the upper half of the mammary gland was necessary. After the breast tissue had been readapted, repositioning the NAC was evaluated. If necessary, the NAC was transferred to the center of the new breast dome by deepithelializing a periareolar crescent of skin opposite to the segmentectomy/quadrantectomy (Figures 7, 8, 9, and 10).

### 2.3. Questionnaires

All patients completed a patient satisfaction questionnaire. The questionnaire contained multiple-choice answers with a 4-point scale about the treated breast compared with the other breast, functional results of the treated breast and arm, and the patients' general satisfaction with the cosmetic outcome.

Some questions were derived from the Body Image Scale (Table 1, questions 2 and 3), the reliability and validity of which have been previously psychometrically tested in various samples [14], as well as from Patterson et al.'s questionnaire [15] (Table 1, question 1) and from the European Organization for Research and Treatment of Life Questionnaire, Breast Cancer Module (EORTC QLQ-BR23; Table 1, questions 9, 11, and 12) [16]. Other questions were created by our study team (Table 1, questions 4–8 and 10). A summary of the questions and the results is shown in Table 1.

### 2.4. Cosmetic Assessment

Each patient was assessed by the same breast surgeon (UH-B). Standardized measurements of the breasts and standardized photographs were obtained for each patient. A five-member panel (three breast surgeons, one plastic surgeon, and one general practitioner) subsequently evaluated the standardized photographs. Each member submitted the evaluation separately according to specific criteria on a rating scale of 1–4 for each patient and each criterion. The evaluation criteria were the appearance of the treated breast, scar, nipple-areola deviation, and volumetric symmetry. For ease of presentation, the median of the assessments was obtained to give a final score for each patient and criterion.

### 2.5. BCCT.core

BCCT.core software (breast cancer conservative treatment cosmetic results), developed by Cardoso's working group, is an objective for evaluating breast asymmetry [17]. The software semiautomatically evaluates a front view image of the breasts and torso. At the beginning, various structures in the photograph, such as the nipples, the sternum, and chest contours, must be marked manually by the examiner. Then objective measures for symmetry such as differences in size, lesions, and scars are computed automatically to provide an overall result. The results are shown on a 4-point scale (1 =* excellent*, 2 =* good*, 3 =* moderate*, and 4 =* poor*).

### 2.6. Correlation between Patient Satisfaction and Patient-, Tumor-, and Treatment-Dependent Factors

The influence of the following patient satisfaction factors was analyzed: age, body mass index (BMI), bra cup size (A, B, C, and D), type of surgical technique (DGR, SE), scar length, resection volume, tumor size, and tumor location (upper inner, upper outer, lower inner quadrant). Moreover, patient satisfaction was correlated to the patients'ratings describing the degree of volume discrepancy between their breasts.

### 2.7. Statistics

We used descriptive statistics to display the characteristics of the patient sample. Differences between the two surgical groups (DGR and SE) were tested for statistical significance using the *χ* ^2^ test and when appropriate Fisher's exact test. *P* < 0.05 was considered significant.

To evaluate the influence of the patient, tumor, and treatment factors on patient satisfaction, the first step was to undertake a univariate analysis using a chi-square test and then the Mann-Whitney *U* test in order to find variables that are relevant to multiple logistic regression. Factors considered significant or nearly significant were selected for multivariate analysis. To obtain a more adequate sample size for multivariate logistic regression, patient satisfaction was combined in two groups. The responses to this variable were categorized with ratings of 1 to 3 (*not at all*,* a little,* and* quite a bit*) grouped as* not very satisfied* and 4 (*very much*) as* very satisfied*. The statistical analysis of the data was performed using SPSS version 11.5 (SPSS, Chicago, IL, USA).

## 3. Results

**Figure 11:**
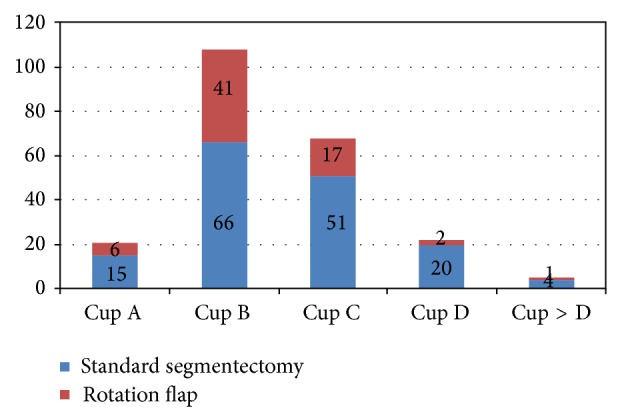
Distribution of brassiere cup size.

The median age was 59 years (range 24 to 87 years, SD 11.04), and the median follow-up period was 24 months (range 7 to 89, SD 19.33); 196 (85%) patients received radiation therapy as part of their treatment. The median body mass index was 25 (range 16 to 42, SD 4.88). Most patients (76%) had a brassiere cup size of B or C. The distribution in the two surgery groups is shown in Figure 11.

In the DGR group, the median tumor size was 23 mm (range 6 to 60 mm, SD 11.52), the median resection volume was 89 g (range 26 to 270 g, SD 49.15), and the median length of the scar was 23 cm (range 7 to 44 cm, SD 6.85). In the segmentectomy group, the median tumor size was 15 mm (range 2 to 55 mm, SD 8.61), the median resection volume was 40 g (range 8 to 215 g, SD 28.07), and the median length of the scar was 5 cm. A total of 22 patients (10%) were administered neoadjuvant therapy. Staging, tumor node metastasis, and axillary intervention are shown in Table 1. More patients in the DGR group than in the SE group had 2 tumors, lymph node metastasis, and therefore dissection of the axillary lymph nodes.

### 3.1. Overall Satisfaction and Cosmetic Assessment

About 92.8% of the patients treated with DGR and 83.5% of the patients treated with SE were very satisfied with the cosmetic appearance of their breasts, showing no significant statistical difference (*P* = 0.189). The feeling of physical attractiveness did not differ between the two surgical treatment groups (*P* = 0.435). The detailed results are shown in Table 2 (questions 1 and 3).

The expert panel judged the aesthetic outcome of the treated breast (scar visibility, position of the NAC, and aesthetic appearance) as* excellent* in 32.2%,* good* in 60.9%,* moderate* in 5.6%, and* poor* in 0.6% of the cases treated with SE. The aesthetic outcome of the treated breast was considered* excellent* in 8.7%,* good* in 63.8%,* moderate* in 24.6%, and* poor* in 0.0% cases treated with DGR. The difference between the 2 groups in the expert panels' evaluation was significant (*P* < 0.001).

### 3.2. BCCT.core

In the patients treated with SE, 10.6% of the breasts were evaluated as* excellent* and 77.0% as* good*. In the DGR group, 4.3% of the breasts were classified as* excellent* and 75.4% as* good*.

More* moderate* results were seen in the patients treated with DGR (18.8%) versus the SE group (10.6%). On a 4-point scale, the differences between the two groups were not significant (*P* = 0.191). Detailed results are shown in Table 3.

### 3.3. Scar

Of the patients, 11.4% who underwent SE stated that the scar was not at all or was only slightly (60.8%) visible compared to 60.3% of the patients in the DGR group, who said that the scar was barely or even not (4.4%) visible. The difference between the patient evaluations was not significant for the 2 groups (*P* = 0.2; Table 2, question 4). The satisfaction of the patients in the SE and DGR groups was high on the 4-point scale and not significantly different between the groups (*P* = 0.435; Table 2, question 2).

In contrast to the patients' view, the expert panel considered the visibility of the scar significantly greater in the rotation flaps group (*P* < 0.0001) as shown in Table 4. In the segmentectomy group, the patient evaluation of the visibility of the scar did not differ from that of the expert panel (*P* = 0.132).

### 3.4. Symmetry of the Breast Volume

In 88.9% of cases, the expert panel stated that there was no (37.3%) or only a little (51.6%) difference in breast volume in the group of patients treated with SE; only 24.4% of the patients in this group estimated the difference in the volume of their breast as* not at all* and 48.1% as* slightly*. On the 4-point scale, the patients estimated the difference in the size of their breasts as larger than the panel of experts in the SE group (*P* = 0.001) as well as in the DGR group (*P* = 0.001) as shown in Table 4. In the patient group treated with SE versus DGR, a significant difference in the perception of the breast volume was notable (*P* = 0.033; Table 2, question 5). Remarkably, this was the only significant difference between the answers of the two surgery groups. Despite this, most of the patients stated that the difference in their breast volume either did not bother or only minimally bothered them in both groups.

### 3.5. Position of the Nipple and Areola

The majority of patients were very satisfied with the position of the nipple-areola complex (74.3% of the SE group, 77.9% of the DGR group; Table 2, question 7). Patient satisfaction with the position of the nipple and areola complex did not differ significantly between the two groups. The experts assessed the position of the nipple-areola complex in the DGR group on a 4-point scale as less satisfying than in the SE group (*P* = 0.001; Table 4).

### 3.6. Morbidity

Of the patients, 70.7% in the SE group and 76.1% in the DGR group reported that they had little or no postoperative pain in the affected breast. On the 4-point scale, no difference between the groups was notable (*P* = 0.751; Table 2, question 8).

The patients in the 2 groups reported no significant difference regarding pain in their breast during the week before the survey, their arm or shoulder, and their scar (Table 2, questions 9–11). At the time of the survey, more than 50% of the patients in both surgical treatment groups stated they had pain in the treated breast. In 17.6% of the SE group and 11.5% of the DGR group, the pain was classified as* moderately severe or higher*; 76.1% of the patients in the SE group and 84.6% of the patients in the DGR group reported that the area of their affected breast was not at all or slightly oversensitive, on a 4-point scale. The difference was not significant (*P* = 0.316). Details are presented in Table 2 (question 12).

### 3.7. Correlation between Patient Satisfaction and Patient-, Tumor-, and Treatment-Dependent Factors

On univariate and multivariate analysis, age (*P* = 0.007) and the patients' ratings of the volume differences between their breasts (*P* = 0.002) significantly were associated with patient satisfaction. Younger age was associated with a higher degree of dissatisfaction. A higher grade of self-perceived volume discrepancy was associated with a higher degree of dissatisfaction.

## 4. Discussion

Oncoplastic breast surgery has been proven to be safe for tumors high in volume and difficult in location in local recurrence and survival rates comparable to conventional breast-conserving therapy [14, 18]. Therefore, we focused on the aesthetic evaluation, especially from the patients' view in comparison to experts', morbidity, and resulting patient satisfaction to gather more information for a better understanding of patient sensation and comfort after the surgical procedures.

The majority of our patients appeared satisfied with the operation independent of the surgical technique. Younger age was significantly associated with a lower degree of satisfaction. Notably, the results of other studies revealed that expert panels gave a lower ranking to the group of older patients [19]. The first could be explained as younger patients have higher expectations than older patients and therefore tend to rank the cosmetic result lower in cases of smaller deviations. According to the expert panels, there are grounds for suspecting that the surgical results are not only rated but also biased by the overall appearance of the women [20].

Overall patient satisfaction with the visibility of the scar was judged the same in both groups. In the SE group, the median scar length was 5 cm, and the patients were as critical concerning the visibility of the scar as the expert panel (*P* = 0.132). In contrast, the patients in the DGR group were less critical about the visibility of their scars than the expert panel in spite of long scars (median 23 cm). The difference was significant (*P* < 0.006). One limitation/disadvantage of DGR, the length of the radial incision with an increasingly visible scarring compared to lumpectomy, has been previously discussed [12]. According to our results this point of view seems to be the expert perception, not the patients'.

Al-Ghazal and Blamey presented data showing no correlation between scar length and satisfaction [21]. Other data demonstrated a clear correlation between these two parameters, but these studies do not all refer to oncoplastic surgery [15].

The evaluation of the position of the nipple-areola complex showed a similar result. The expert panel assessed the position of the nipple-areola complex in the DGR group as significantly less satisfying than in the SE group. In contrast, patient satisfaction was the same in the groups.

The evaluation of the difference in breast volume showed a significant discrepancy between the patients in the 2 treatment groups: the patients with DGR perceived a significantly higher difference in volume between their breasts than the patients with SE.

**Figure 12:**
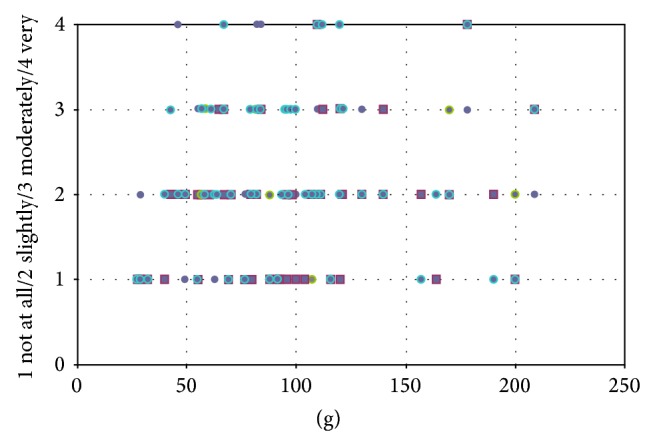
Dermoglandular rotation flap: correlation between resection volume and difference in the breast volume (subjective rating of the expert panel).

This result can be explained due to the higher median resection volume in the rotation flaps group. The risk of asymmetry in cases of higher resection volumes in general increases but this does not mean that a high resection volume means a big difference in the overall breast volume in every single case based on subjective expert panel assessment (as seen in Figure 12). We detected that in the DGR group despite higher resection volumes the transposition of soft tissue from the lateral subaxillary region reduced the expected asymmetry in some cases.

The majority of patients in both groups were not bothered by the discrepancy (see Table 1, question 6), but a minority of patients, 20.4% of the patients in the SE group and 10.3% of the patients in the DGR group, felt moderately or extremely bothered by the perceived difference in breast volume. This is proven by the negative impact between the degree of asymmetry and patient satisfaction. Remarkably, the patients in both treatments rated the discrepancy in breast volume on a 4-point scale significantly higher than the expert panel. These results confirmed other investigations, in which good patients' evaluation attends critical assessment of the discrepancy in breast volume [16, 22].

In this context, one limitation of DGR may be that, depending on the tumor location and size, a different surgical technique must be chosen for symmetrizing procedures on the contralateral side, if desired by the patient. In cases of the necessity to deepithelize a periareolar crescent, we recommend a periareolar approach in performing contralateral mammaplasty.

At the time of the survey, more than 50% of the patients in both surgical treatment groups reported they suffered from pain in the treated breast. Chronic pain is a common problem after breast-conserving therapy, as 25–60% of patients complain about it [23–25].

There was no significant difference between the 2 surgical groups regarding the pain in the breast, so the hypothesis that larger tissue trauma leads to increased postoperative or chronic pain cannot be confirmed. Obesity is under discussion as a possible risk factor for pain; however, surgical techniques have not been shown to play a predominant role in postoperative pain [26].

## 5. Conclusion

DGR for managing tumors in the upper inner, lower inner, and upper outer quadrant delivers high patient satisfaction and similar comfort after breast-conserving therapy compared to SE despite a higher median resection volume, a significantly longer scar, and a higher grade of asymmetry perceived by the patients and the expert panel. Younger age and a higher degree of perceived volume discrepancy between the breasts had a negative impact on patient satisfaction independent of the type of surgery. The perception of the grade of asymmetry of the breast and the position of the NAC was significantly different between the patients and the expert panel in both treatment groups, whereas the visibility of the scars was different only in the DGR group.

## Figures and Tables

**Table 1:** Summary of staging and axillary intervention.

	Segmentectomy group (*n* = 161)		Rotation flap group (*n* = 69)		Total	
T1	102	63.4%	19	27.5%	121	52.6%
T2	29	18.0%	29	42.0%	58	25.2%
T3	0	0.0%	1	1.4%	1	0.4%
pTis	18	11.2%	10	14.5%	28	12.2%
yT0	2	1.2%	4	5.8%	6	2.6%
yT1	8	5.0%	4	5.8%	12	5.2%
yT2	1	0.6%	0	0.0%	1	0.4%
yT3	1	0.6%	0	0.0%	1	0.4%
ypTis	0	0.0%	2	2.9%	2	0.9%
Total cases	**161**	**100.0%**	**69**	**100.0%**	**230**	**100.0%**

Axillary dissection	33	20.5%	21	30.4%	54	23.5%
Sentinel	102	63.4%	37	53.6%	139	60.4%
None	15	9.3%	8	11.6%	23	10.0%
Missing	11	6.8%	3	4.3%	14	6.1%

N0	118	73.3%	41	59.4%	159	69.1%
N1	17	10.6%	12	17.4%	29	12.6%
N2	4	2.5%	4	5.8%	8	3.5%
N3	1	0.6%	2	2.9%	3	1.3%
N*x*	21	13.0%	10	14.5%	31	13.5%
Total cases	**161**	**100.0%**	**69**	**100.0%**	**230**	**100.0%**

**Table 2:** Patients' answers to the main questions and significance of the differences between the segmentectomy group and rotation flap group.

Question	Group segmentectomy *n* = 161					Group rotation flap *n* = 69					*P* value
	1	2	3	4	Total	1	2	3	4	Total	
(1) How satisfied are you with the cosmetic appearance of your breast?	0.6%	4.4%	11.4%	83.5%	158	0.0%	0.0%	7.2%	92.8%	69	0.189
(2) Have you been dissatisfied with the appearance of your scar?	70.9%	17.7%	7.6%	3.8%	158	72.1%	19.1%	8.8%	0.0%	68	0.435
(3) Have you felt physically less attractive as a result of your disease or treatment?	74.8%	11.0%	9.7%	4.5%	155	77.3%	15.2%	7.6%	0.0%	66	0.272
(4) How visible are the scars?	11.4%	60.8%	20.9%	7.0%	158	4.4%	60.3%	22.1%	13.2%	68	0.200
(5) How different is the volume of your treated breast from your other breast?	24.4%	48.1%	21.2%	6.4%	156	15.9%	52.2%	14.5%	17.4%	69	0.033
(6) Does the difference disturb you?	56.8%	22.9%	15.3%	5.1%	118	72.4%	17.2%	6.9%	3.4%	58	0.210
(7) Are you satisfied with the position of your nipple and areola?	12.9%	1.4%	11.4%	74.3%	140	7.4%	7.4%	7.4%	77.9%	68	0.076
(8) Did you have pain in your affected breast immediately after the operation?	29.3%	41.4%	22.9%	6.4%	157	35.8%	40.3%	19.4%	4.5%	67	0.751
(9) Have you had any pain in the area of your affected breast during the past week?	47.8%	34.6%	15.1%	2.5%	159	44.9%	43.5%	10.1%	1.4%	69	0.528
(10) Do you have pain in your scar?	64.3%	29.9%	3.8%	1.9%	157	59.4%	29.0%	11.6%	0.0%	69	0.103
(11) Do you have any pain in your arm or shoulder?	61.4%	19.0%	14.6%	5.1%	158	57.4%	23.5%	14.7%	4.4%	68	0.884
(12) Was the area of your affected breast oversensitive?	43.40	32.7	18.24	5.66	159	42.03	42.03	14.49	1.45	69	0.316

^*^1 = not at all. 2 = slightly. 3 = moderately. 4 = very.

**Table 3:** BCCT.core results.

Group segmentectomy *n* = 161					Group rotation flap *n* = 69					*P* value
Excellent	Good	Moderate	Poor	Total	Excellent	Good	Moderate	Poor	Total	
17	124	17	3	161	3	52	13	1	69	0.191
10.6%	77.0%	10.6%	1.9%	100.0%	4.3%	75.4%	18.8%	1.4%	100.0%	

**Table 4:** Comparison of the expert panel and patient answers to the questions.

	Scale	Group segmentectomy					*P* value	Group rotation flap					*P* value
		1	2	3	4	Total		1	2	3	4	Total	
Patients	How visible are the scars?	11.4%	60.8%	20.9%	7.0%	158	0.132	4.4%	60.3%	22.1%	13.2%	68	0.006
Expert panel	How visible are the scars?	13.7%	59.6%	24.8%	1.9%	161		0%	37.7%	44.9%	17.4%	69	
Patients	How different is the volume of your treated breast from your other breast?	24.4%	48.1%	21.2%	6.4%	156	0.001	15.9%	52.2%	14.5%	17.4%	69	0.001
Expert panel	How different is the volume of the treated breast from the other breast?	37.3%	51.6%	9.9%	1.2%	161		24.6%	42.0%	31.9%	1.4%	69	
Patients	Are you satisfied with the position of your nipple and areola?	12.9%	1.4%	11.4%	74.3%	140	0.000	7.4%	7.4%	7.4%	77.9%	68	0.000
Expert panel	How satisfying is the position of the nipple and areola?	0%	2.5%	28.0%	69.6%	161		0%	5.8%	50.7%	43.5%	69	

^*^1 = not at all. 2 = slightly visible. 3 = moderately visible. 4 = very visible.
